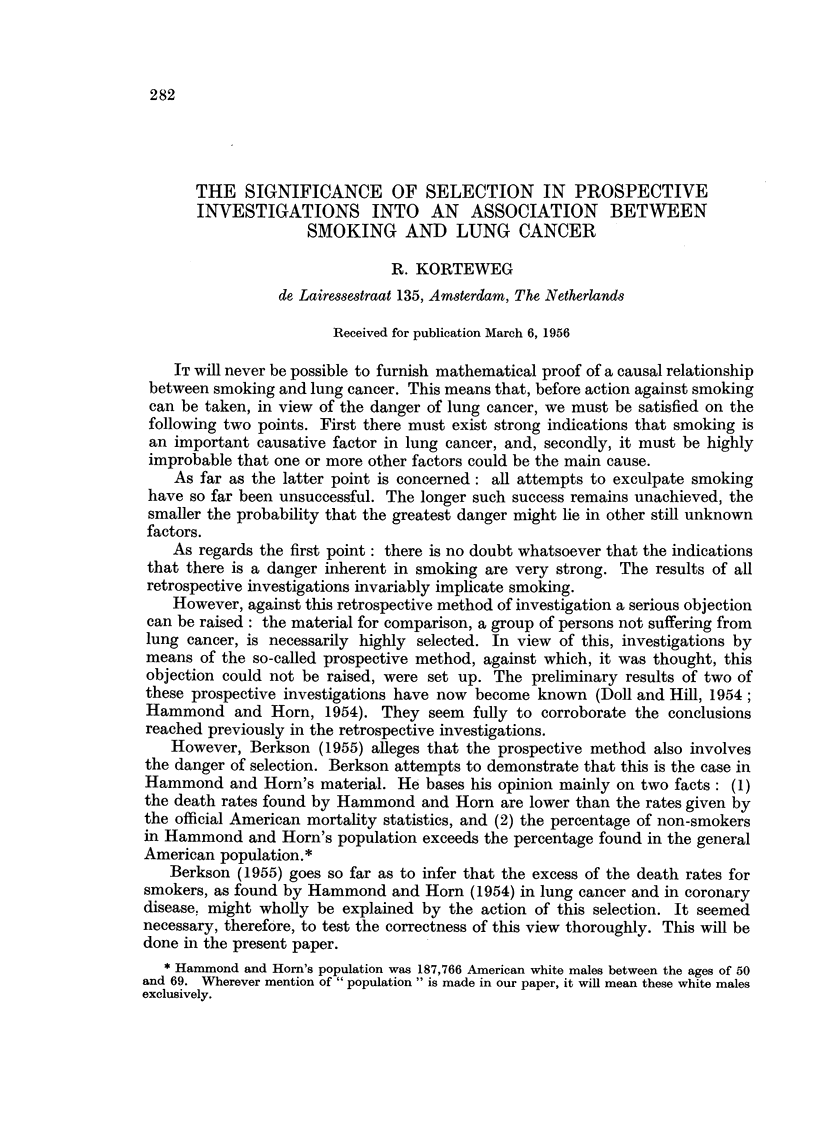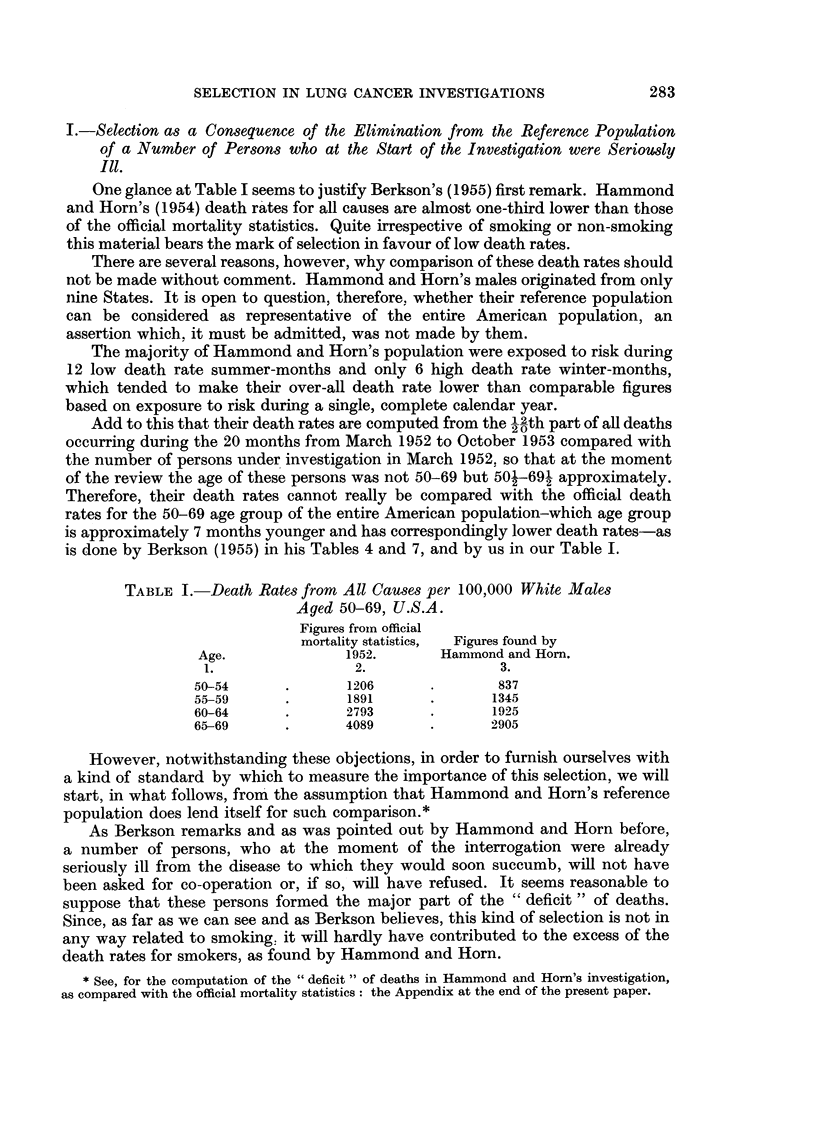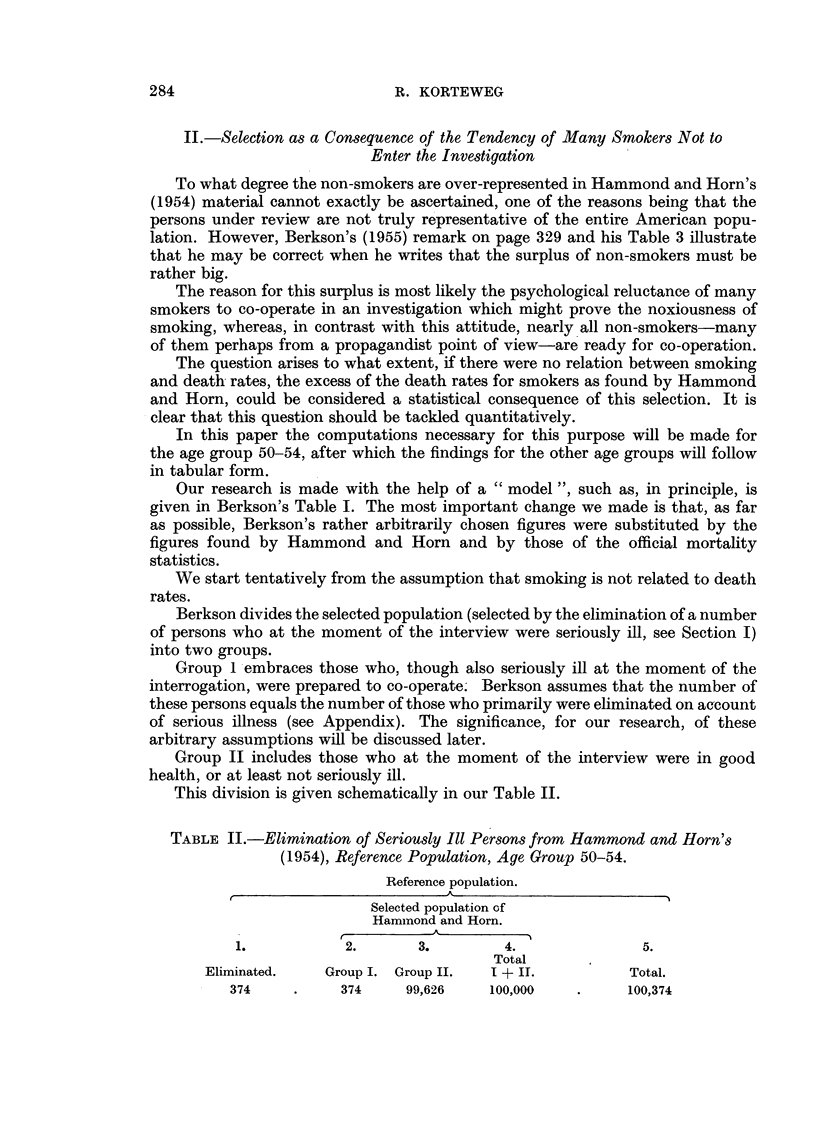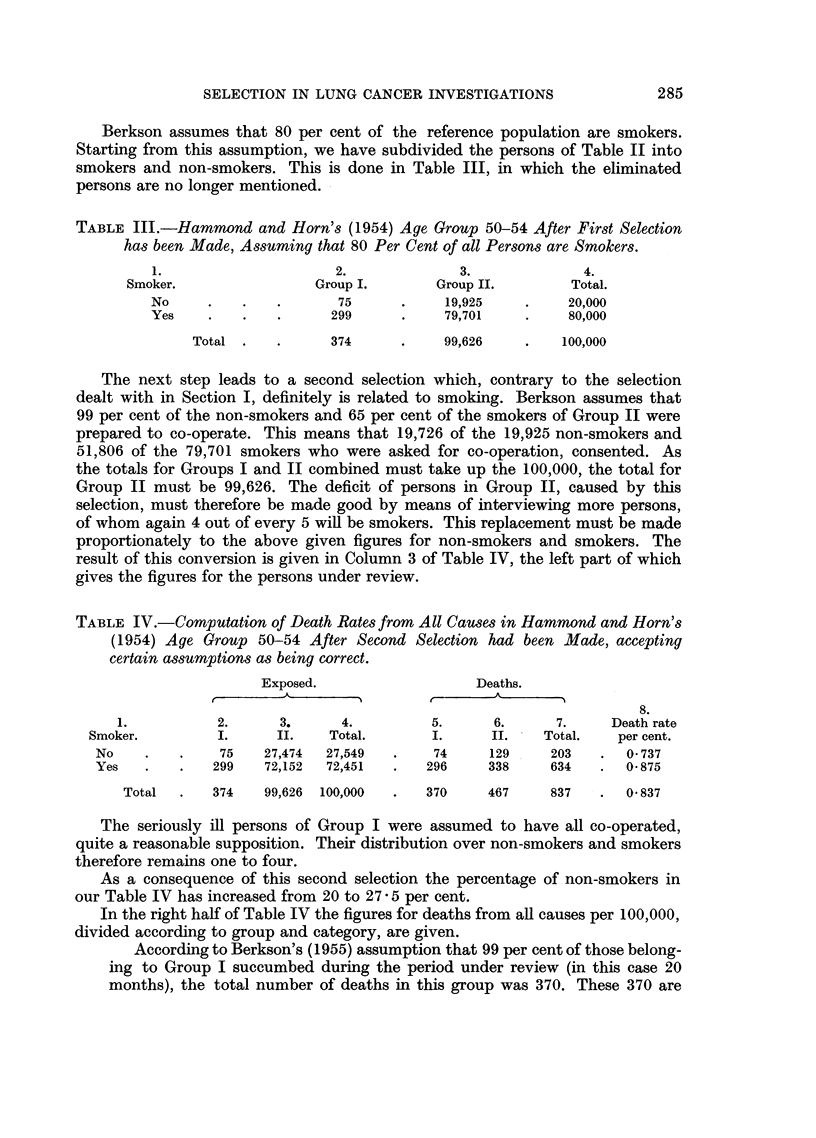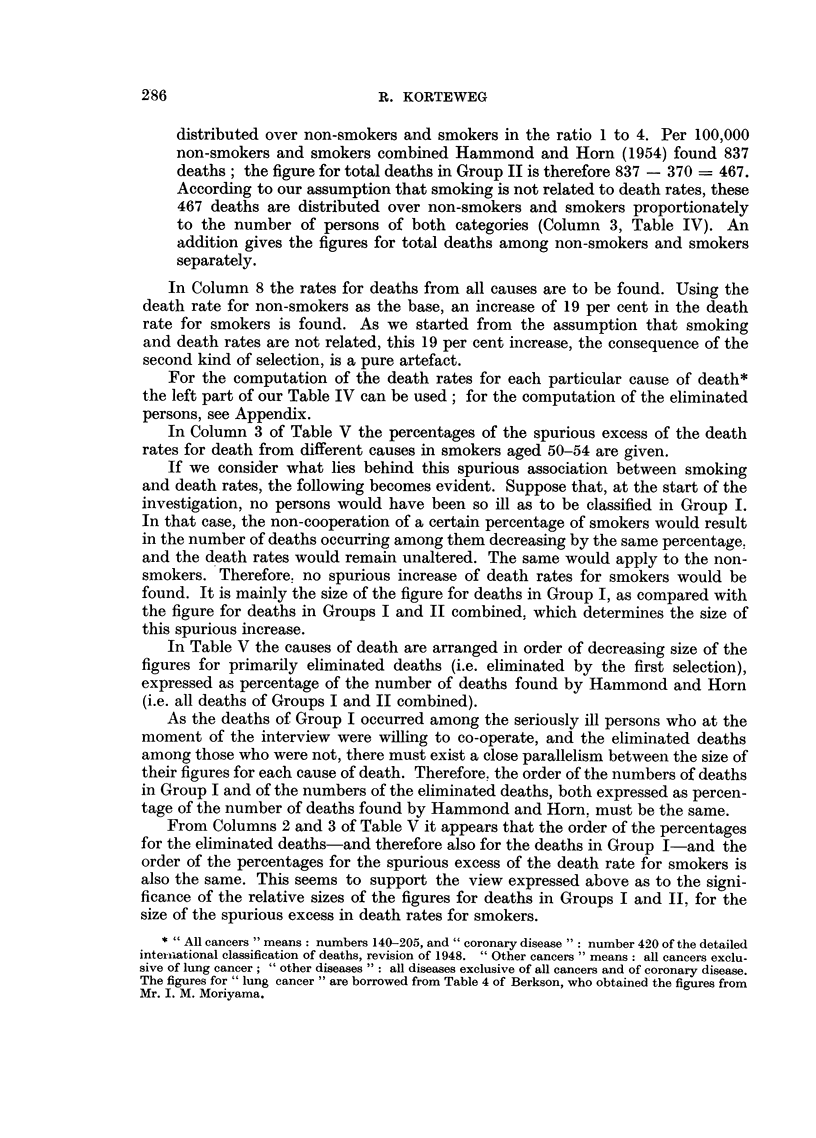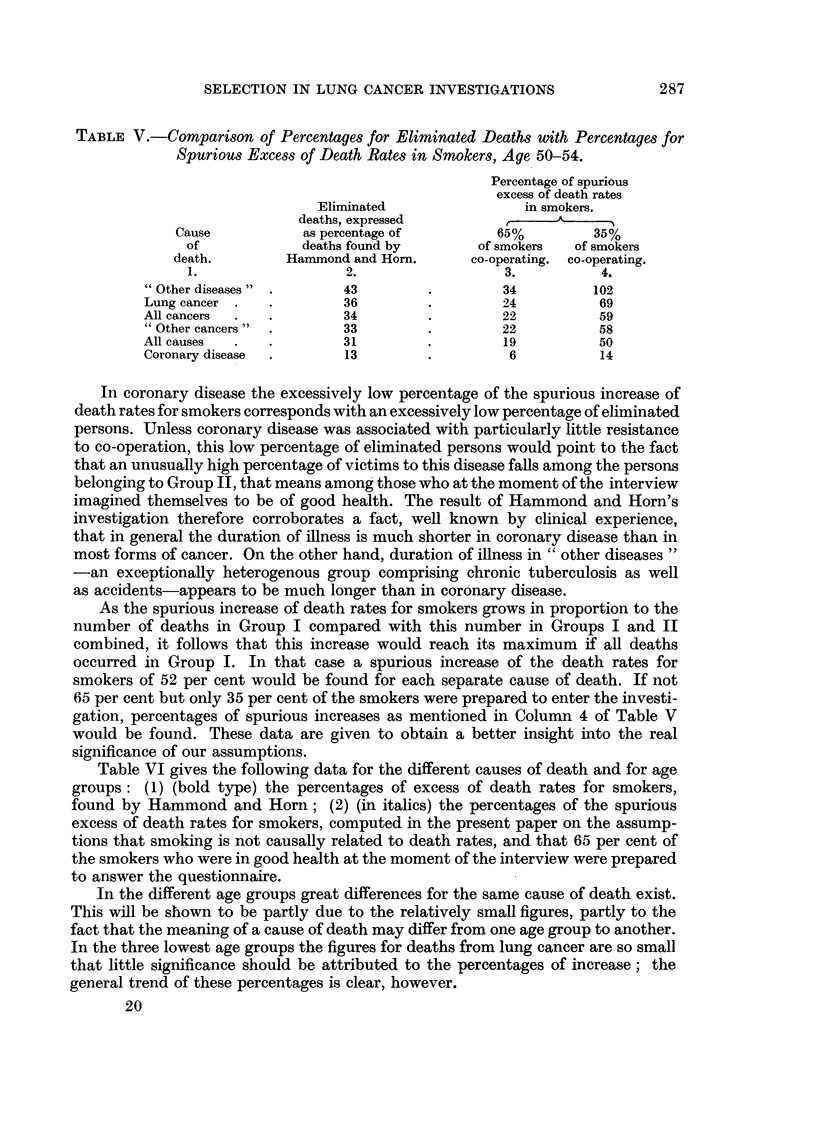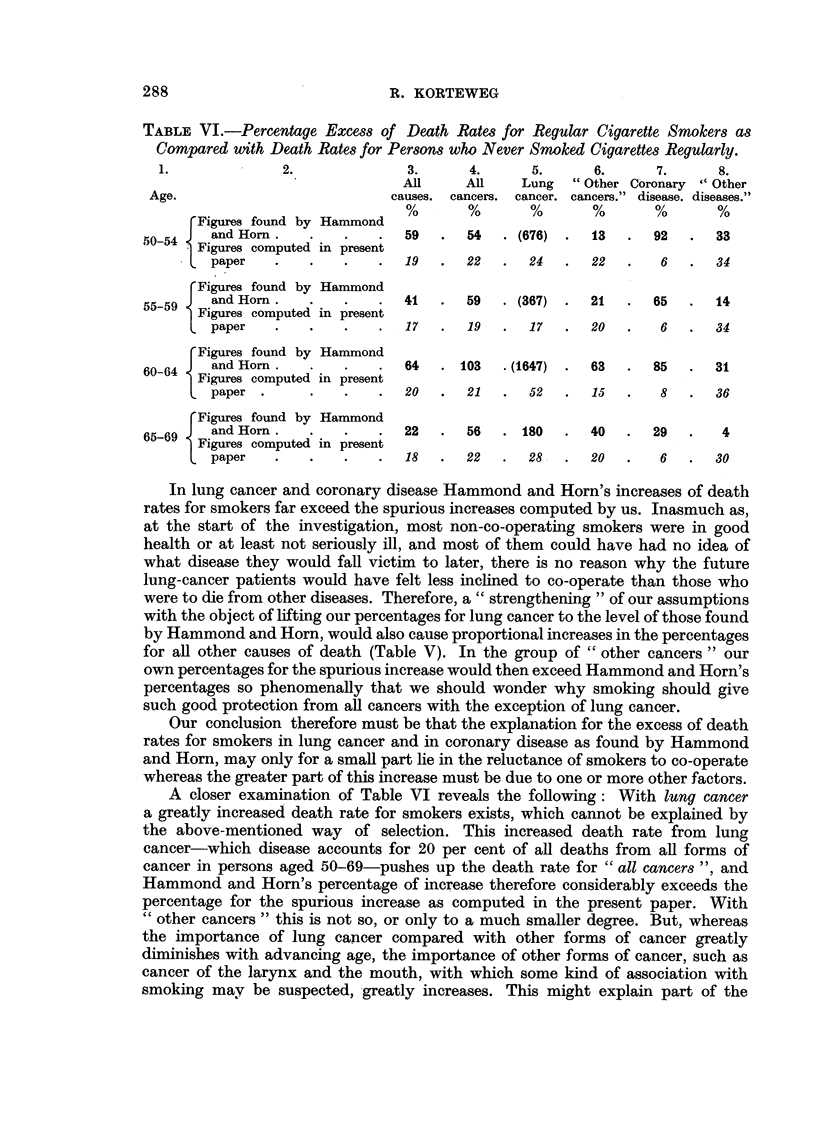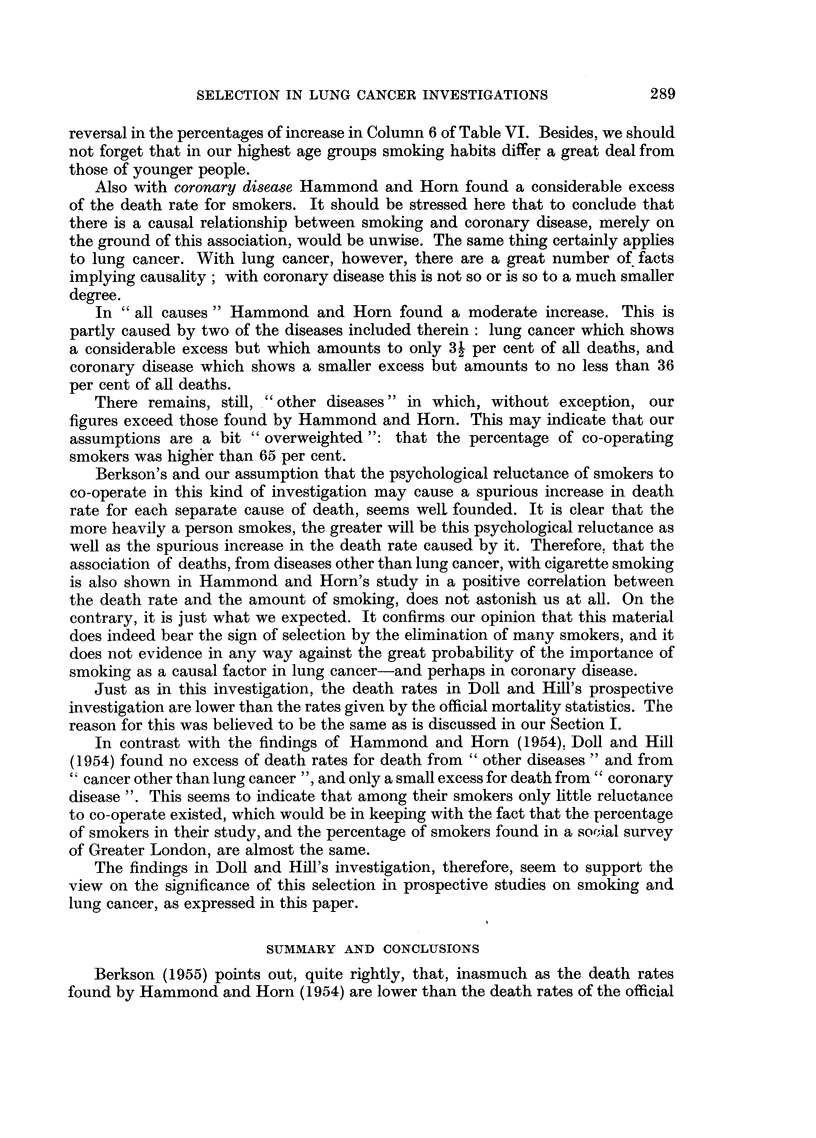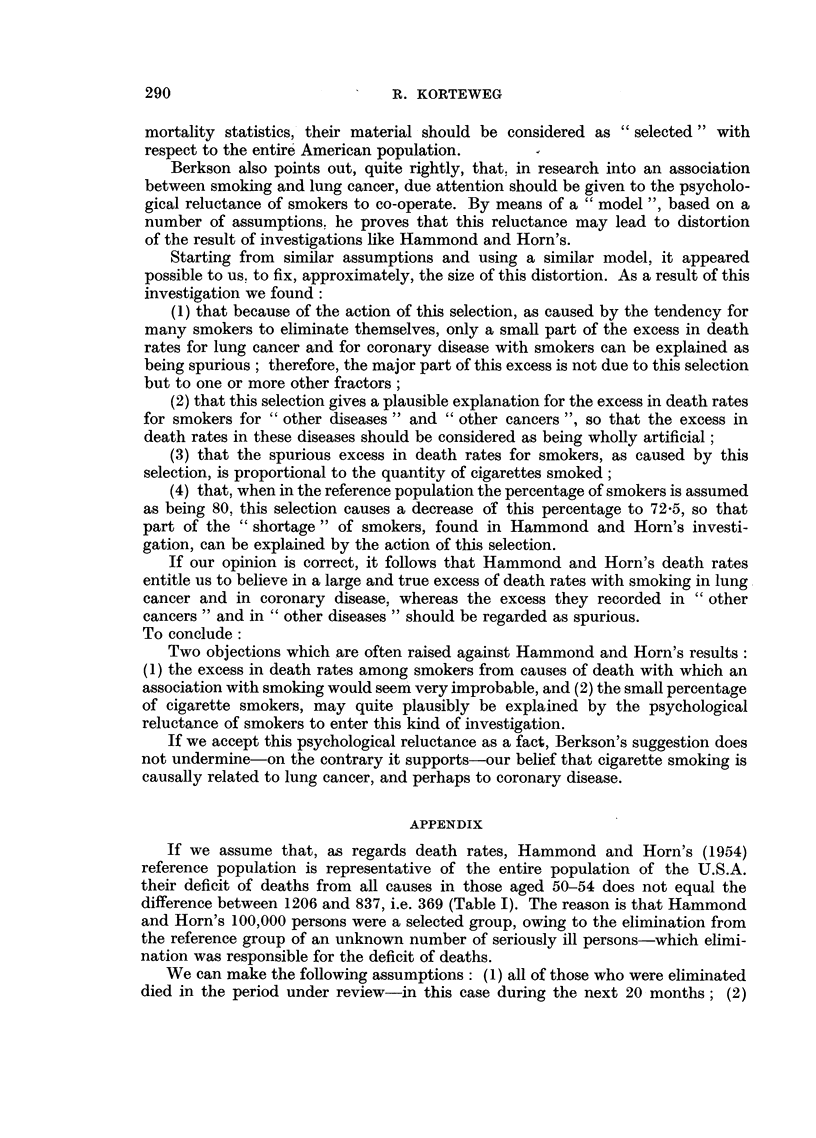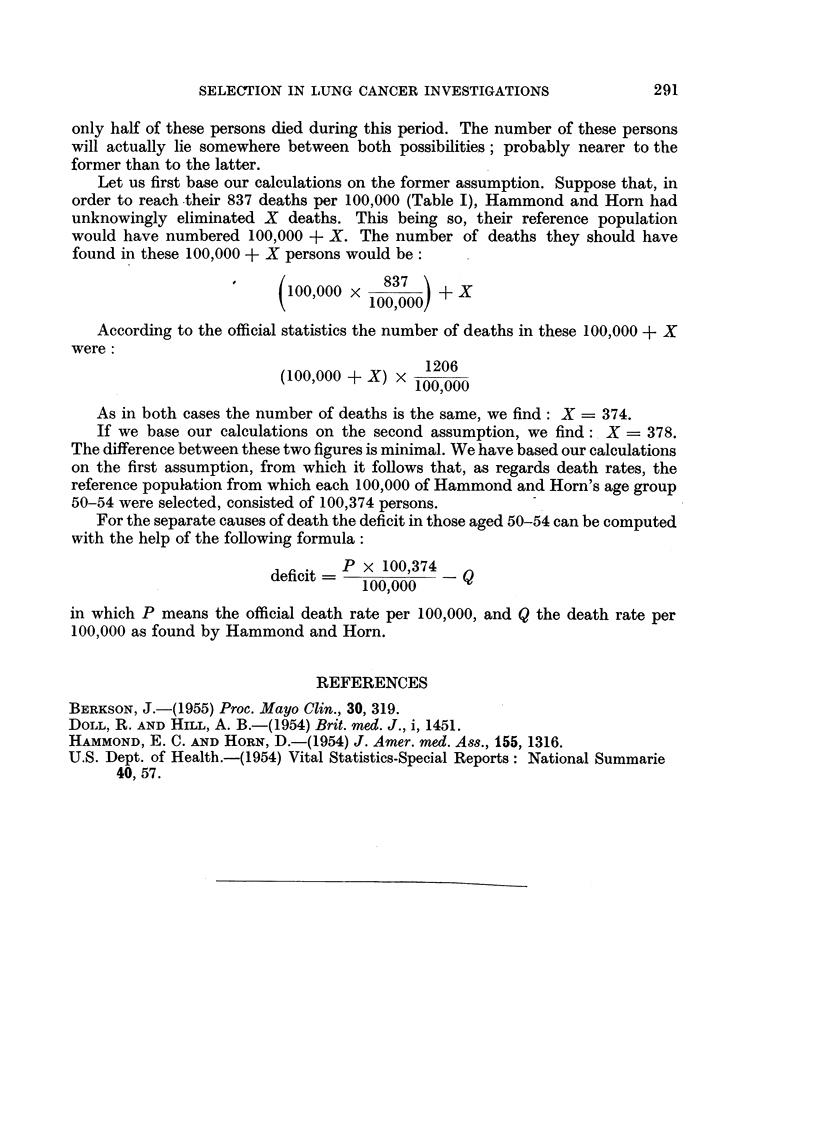# The Significance of Selection in Prospective Investigations into an Association between Smoking and Lung Cancer

**DOI:** 10.1038/bjc.1956.32

**Published:** 1956-06

**Authors:** R. Korteweg


### THE SIGNIFICANCE OF SELECTION IN PROSPECTIVE INVESTIGATIONS INTO AN ASSOCIATION BETWEEN SMOKING AND LUNG CANCER

R. KORTEWEG

*de Lairessestraat* 135, *Amsterdam*, *The Netherlands*

Received for publication March 6, 1956

It will never be possible to furnish mathematical proof of a causal relationship between smoking and lung cancer. This means that, before action against smoking can be taken, in view of the danger of lung cancer, we must be satisfied on the following two points. First there must exist strong indications that smoking is an important causative factor in lung cancer, and, secondly, it must be highly improbable that one or more other factors could be the main cause.

As far as the latter point is concerned: all attempts to exculpate smoking have so far been unsuccessful. The longer such success remains unachieved, the smaller the probability that the greatest danger might lie in other still unknown factors.

As regards the first point: there is no doubt whatsoever that the indications that there is a danger inherent in smoking are very strong. The results of all retrospective investigations invariably implicate smoking.

However, against this retrospective method of investigation a serious objection can be raised: the material for comparison, a group of persons not suffering from lung cancer, is necessarily highly selected. In view of this, investigations by means of the so-called prospective method, against which, it was thought, this objection could not be raised, were set up. The preliminary results of two of these prospective investigations have now become known (Doll and Hill, 1954; Hammond and Horn, 1954). They seem fully to corroborate the conclusions reached previously in the retrospective investigations.

However, Berkson (1955) alleges that the prospective method also involves the danger of selection. Berkson attempts to demonstrate that this is the case in Hammond and Horn's material. He bases his opinion mainly on two facts: (1) the death rates found by Hammond and Horn are lower than the rates given by the official American mortality statistics, and (2) the percentage of non-smokers in Hammond and Horn's population exceeds the percentage found in the general American population.*

Berkson (1955) goes so far as to infer that the excess of the death rates for smokers, as found by Hammond and Horn (1954) in lung cancer and in coronary disease, might wholly be explained by the action of this selection. It seemed necessary, therefore, to test the correctness of this view thoroughly. This will be done in the present paper.

* Hammond and Horn's population was 187,766 American white males between the ages of 50 and 69. Wherever mention of " population " is made in our paper, it will mean these white males exclusively.

I.—*Selection as a Consequence of the Elimination from the Reference Population of a Number of Persons who at the Start of the Investigation were Seriously Ill.*

One glance at Table I seems to justify Berkson's (1955) first remark. Hammond and Horn's (1954) death rates for all causes are almost one-third lower than those of the official mortality statistics. Quite irrespective of smoking or non-smoking this material bears the mark of selection in favour of low death rates.

There are several reasons, however, why comparison of these death rates should not be made without comment. Hammond and Horn's males originated from only nine States. It is open to question, therefore, whether their reference population can be considered as representative of the entire American population, an assertion which, it must be admitted, was not made by them.

The majority of Hammond and Horn's population were exposed to risk during 12 low death rate summer-months and only 6 high death rate winter-months, which tended to make their over-all death rate lower than comparable figures based on exposure to risk during a single, complete calendar year.

Add to this that their death rates are computed from the $\frac{12}{20}$th part of all deaths occurring during the 20 months from March 1952 to October 1953 compared with the number of persons under investigation in March 1952, so that at the moment of the review the age of these persons was not 50–69 but $50\frac{1}{2}$–$69\frac{1}{2}$ approximately. Therefore, their death rates cannot really be compared with the official death rates for the 50–69 age group of the entire American population–which age group is approximately 7 months younger and has correspondingly lower death rates—as is done by Berkson (1955) in his Tables 4 and 7, and by us in our Table I.

TABLE I.—*Death Rates from All Causes per* 100,000 *White Males Aged* 50–69, *U.S.A.*

| Age. 1. | Figures from official mortality statistics, 1952. 2. | Figures found by Hammond and Horn. 3. |
|---|---|---|
| 50–54 | 1206 | 837 |
| 55–59 | 1891 | 1345 |
| 60–64 | 2793 | 1925 |
| 65–69 | 4089 | 2905 |

However, notwithstanding these objections, in order to furnish ourselves with a kind of standard by which to measure the importance of this selection, we will start, in what follows, from the assumption that Hammond and Horn's reference population does lend itself for such comparison.*

As Berkson remarks and as was pointed out by Hammond and Horn before, a number of persons, who at the moment of the interrogation were already seriously ill from the disease to which they would soon succumb, will not have been asked for co-operation or, if so, will have refused. It seems reasonable to suppose that these persons formed the major part of the " deficit " of deaths. Since, as far as we can see and as Berkson believes, this kind of selection is not in any way related to smoking, it will hardly have contributed to the excess of the death rates for smokers, as found by Hammond and Horn.

* See, for the computation of the " deficit " of deaths in Hammond and Horn's investigation, as compared with the official mortality statistics : the Appendix at the end of the present paper.

### II.—*Selection as a Consequence of the Tendency of Many Smokers Not to Enter the Investigation*

To what degree the non-smokers are over-represented in Hammond and Horn's (1954) material cannot exactly be ascertained, one of the reasons being that the persons under review are not truly representative of the entire American population. However, Berkson's (1955) remark on page 329 and his Table 3 illustrate that he may be correct when he writes that the surplus of non-smokers must be rather big.

The reason for this surplus is most likely the psychological reluctance of many smokers to co-operate in an investigation which might prove the noxiousness of smoking, whereas, in contrast with this attitude, nearly all non-smokers—many of them perhaps from a propagandist point of view—are ready for co-operation.

The question arises to what extent, if there were no relation between smoking and death rates, the excess of the death rates for smokers as found by Hammond and Horn, could be considered a statistical consequence of this selection. It is clear that this question should be tackled quantitatively.

In this paper the computations necessary for this purpose will be made for the age group 50–54, after which the findings for the other age groups will follow in tabular form.

Our research is made with the help of a " model ", such as, in principle, is given in Berkson's Table I. The most important change we made is that, as far as possible, Berkson's rather arbitrarily chosen figures were substituted by the figures found by Hammond and Horn and by those of the official mortality statistics.

We start tentatively from the assumption that smoking is not related to death rates.

Berkson divides the selected population (selected by the elimination of a number of persons who at the moment of the interview were seriously ill, see Section I) into two groups.

Group 1 embraces those who, though also seriously ill at the moment of the interrogation, were prepared to co-operate. Berkson assumes that the number of these persons equals the number of those who primarily were eliminated on account of serious illness (see Appendix). The significance, for our research, of these arbitrary assumptions will be discussed later.

Group II includes those who at the moment of the interview were in good health, or at least not seriously ill.

This division is given schematically in our Table II.

TABLE II.—*Elimination of Seriously Ill Persons from Hammond and Horn's* (1954), *Reference Population, Age Group* 50–54.

| Reference population. | | | | |
|---|---|---|---|---|
| | Selected population of Hammond and Horn. | | | |
| 1. | 2. | 3. | 4. | 5. |
| Eliminated. | Group I. | Group II. | Total I + II. | Total. |
| 374 | 374 | 99,626 | 100,000 | 100,374 |

Berkson assumes that 80 per cent of the reference population are smokers. Starting from this assumption, we have subdivided the persons of Table II into smokers and non-smokers. This is done in Table III, in which the eliminated persons are no longer mentioned.

TABLE III.—*Hammond and Horn's* (1954) *Age Group* 50–54 *After First Selection has been Made, Assuming that* 80 *Per Cent of all Persons are Smokers.*

| 1. Smoker. | 2. Group I. | 3. Group II. | 4. Total. |
|---|---|---|---|
| No | 75 | 19,925 | 20,000 |
| Yes | 299 | 79,701 | 80,000 |
| Total | 374 | 99,626 | 100,000 |

The next step leads to a second selection which, contrary to the selection dealt with in Section I, definitely is related to smoking. Berkson assumes that 99 per cent of the non-smokers and 65 per cent of the smokers of Group II were prepared to co-operate. This means that 19,726 of the 19,925 non-smokers and 51,806 of the 79,701 smokers who were asked for co-operation, consented. As the totals for Groups I and II combined must take up the 100,000, the total for Group II must be 99,626. The deficit of persons in Group II, caused by this selection, must therefore be made good by means of interviewing more persons, of whom again 4 out of every 5 will be smokers. This replacement must be made proportionately to the above given figures for non-smokers and smokers. The result of this conversion is given in Column 3 of Table IV, the left part of which gives the figures for the persons under review.

TABLE IV.—*Computation of Death Rates from All Causes in Hammond and Horn's* (1954) *Age Group* 50–54 *After Second Selection had been Made, accepting certain assumptions as being correct.*

| | Exposed. | | | Deaths. | | | |
|---|---|---|---|---|---|---|---|
| 1. Smoker. | 2. I. | 3. II. | 4. Total. | 5. I. | 6. II. | 7. Total. | 8. Death rate per cent. |
| No | 75 | 27,474 | 27,549 | 74 | 129 | 203 | 0·737 |
| Yes | 299 | 72,152 | 72,451 | 296 | 338 | 634 | 0·875 |
| Total | 374 | 99,626 | 100,000 | 370 | 467 | 837 | 0·837 |

The seriously ill persons of Group I were assumed to have all co-operated, quite a reasonable supposition. Their distribution over non-smokers and smokers therefore remains one to four.

As a consequence of this second selection the percentage of non-smokers in our Table IV has increased from 20 to 27·5 per cent.

In the right half of Table IV the figures for deaths from all causes per 100,000, divided according to group and category, are given.

According to Berkson's (1955) assumption that 99 per cent of those belonging to Group I succumbed during the period under review (in this case 20 months), the total number of deaths in this group was 370. These 370 are distributed over non-smokers and smokers in the ratio 1 to 4. Per 100,000 non-smokers and smokers combined Hammond and Horn (1954) found 837 deaths ; the figure for total deaths in Group II is therefore 837 — 370 = 467. According to our assumption that smoking is not related to death rates, these 467 deaths are distributed over non-smokers and smokers proportionately to the number of persons of both categories (Column 3, Table IV). An addition gives the figures for total deaths among non-smokers and smokers separately.

In Column 8 the rates for deaths from all causes are to be found. Using the death rate for non-smokers as the base, an increase of 19 per cent in the death rate for smokers is found. As we started from the assumption that smoking and death rates are not related, this 19 per cent increase, the consequence of the second kind of selection, is a pure artefact.

For the computation of the death rates for each particular cause of death* the left part of our Table IV can be used ; for the computation of the eliminated persons, see Appendix.

In Column 3 of Table V the percentages of the spurious excess of the death rates for death from different causes in smokers aged 50–54 are given.

If we consider what lies behind this spurious association between smoking and death rates, the following becomes evident. Suppose that, at the start of the investigation, no persons would have been so ill as to be classified in Group I. In that case, the non-cooperation of a certain percentage of smokers would result in the number of deaths occurring among them decreasing by the same percentage, and the death rates would remain unaltered. The same would apply to the non-smokers. Therefore, no spurious increase of death rates for smokers would be found. It is mainly the size of the figure for deaths in Group I, as compared with the figure for deaths in Groups I and II combined, which determines the size of this spurious increase.

In Table V the causes of death are arranged in order of decreasing size of the figures for primarily eliminated deaths (i.e. eliminated by the first selection), expressed as percentage of the number of deaths found by Hammond and Horn (i.e. all deaths of Groups I and II combined).

As the deaths of Group I occurred among the seriously ill persons who at the moment of the interview were willing to co-operate, and the eliminated deaths among those who were not, there must exist a close parallelism between the size of their figures for each cause of death. Therefore, the order of the numbers of deaths in Group I and of the numbers of the eliminated deaths, both expressed as percentage of the number of deaths found by Hammond and Horn, must be the same.

From Columns 2 and 3 of Table V it appears that the order of the percentages for the eliminated deaths—and therefore also for the deaths in Group I—and the order of the percentages for the spurious excess of the death rate for smokers is also the same. This seems to support the view expressed above as to the significance of the relative sizes of the figures for deaths in Groups I and II, for the size of the spurious excess in death rates for smokers.

* " All cancers " means : numbers 140–205, and " coronary disease " : number 420 of the detailed international classification of deaths, revision of 1948. " Other cancers " means : all cancers exclusive of lung cancer ; " other diseases " : all diseases exclusive of all cancers and of coronary disease. The figures for " lung cancer " are borrowed from Table 4 of Berkson, who obtained the figures from Mr. I. M. Moriyama.

TABLE V.—*Comparison of Percentages for Eliminated Deaths with Percentages for Spurious Excess of Death Rates in Smokers, Age* 50–54.

| Cause of death. 1. | Eliminated deaths, expressed as percentage of deaths found by Hammond and Horn. 2. | Percentage of spurious excess of death rates in smokers. 65% of smokers co-operating. 3. | Percentage of spurious excess of death rates in smokers. 35% of smokers co-operating. 4. |
|---|---|---|---|
| " Other diseases " | 43 | 34 | 102 |
| Lung cancer | 36 | 24 | 69 |
| All cancers | 34 | 22 | 59 |
| " Other cancers " | 33 | 22 | 58 |
| All causes | 31 | 19 | 50 |
| Coronary disease | 13 | 6 | 14 |

In coronary disease the excessively low percentage of the spurious increase of death rates for smokers corresponds with an excessively low percentage of eliminated persons. Unless coronary disease was associated with particularly little resistance to co-operation, this low percentage of eliminated persons would point to the fact that an unusually high percentage of victims to this disease falls among the persons belonging to Group II, that means among those who at the moment of the interview imagined themselves to be of good health. The result of Hammond and Horn's investigation therefore corroborates a fact, well known by clinical experience, that in general the duration of illness is much shorter in coronary disease than in most forms of cancer. On the other hand, duration of illness in " other diseases " —an exceptionally heterogenous group comprising chronic tuberculosis as well as accidents—appears to be much longer than in coronary disease.

As the spurious increase of death rates for smokers grows in proportion to the number of deaths in Group I compared with this number in Groups I and II combined, it follows that this increase would reach its maximum if all deaths occurred in Group I. In that case a spurious increase of the death rates for smokers of 52 per cent would be found for each separate cause of death. If not 65 per cent but only 35 per cent of the smokers were prepared to enter the investigation, percentages of spurious increases as mentioned in Column 4 of Table V would be found. These data are given to obtain a better insight into the real significance of our assumptions.

Table VI gives the following data for the different causes of death and for age groups : (1) (bold type) the percentages of excess of death rates for smokers, found by Hammond and Horn ; (2) (in italics) the percentages of the spurious excess of death rates for smokers, computed in the present paper on the assumptions that smoking is not causally related to death rates, and that 65 per cent of the smokers who were in good health at the moment of the interview were prepared to answer the questionnaire.

In the different age groups great differences for the same cause of death exist. This will be shown to be partly due to the relatively small figures, partly to the fact that the meaning of a cause of death may differ from one age group to another. In the three lowest age groups the figures for deaths from lung cancer are so small that little significance should be attributed to the percentages of increase ; the general trend of these percentages is clear, however.

TABLE VI.—*Percentage Excess of Death Rates for Regular Cigarette Smokers as Compared with Death Rates for Persons who Never Smoked Cigarettes Regularly.*

| 1. Age. | 2. | 3. All causes. % | 4. All cancers. % | 5. Lung cancer. % | 6. " Other cancers." % | 7. Coronary disease. % | 8. " Other diseases." % |
|---|---|---|---|---|---|---|---|
| 50–54 | Figures found by Hammond and Horn | **59** | **54** | **(676)** | **13** | **92** | **33** |
| | Figures computed in present paper | *19* | *22* | *24* | *22* | *6* | *34* |
| 55–59 | Figures found by Hammond and Horn | **41** | **59** | **(367)** | **21** | **65** | **14** |
| | Figures computed in present paper | *17* | *19* | *17* | *20* | *6* | *34* |
| 60–64 | Figures found by Hammond and Horn | **64** | **103** | **(1647)** | **63** | **85** | **31** |
| | Figures computed in present paper | *20* | *21* | *52* | *15* | *8* | *36* |
| 65–69 | Figures found by Hammond and Horn | **22** | **56** | **180** | **40** | **29** | **4** |
| | Figures computed in present paper | *18* | *22* | *28* | *20* | *6* | *30* |

In lung cancer and coronary disease Hammond and Horn's increases of death rates for smokers far exceed the spurious increases computed by us. Inasmuch as, at the start of the investigation, most non-co-operating smokers were in good health or at least not seriously ill, and most of them could have had no idea of what disease they would fall victim to later, there is no reason why the future lung-cancer patients would have felt less inclined to co-operate than those who were to die from other diseases. Therefore, a " strengthening " of our assumptions with the object of lifting our percentages for lung cancer to the level of those found by Hammond and Horn, would also cause proportional increases in the percentages for all other causes of death (Table V). In the group of " other cancers " our own percentages for the spurious increase would then exceed Hammond and Horn's percentages so phenomenally that we should wonder why smoking should give such good protection from all cancers with the exception of lung cancer.

Our conclusion therefore must be that the explanation for the excess of death rates for smokers in lung cancer and in coronary disease as found by Hammond and Horn, may only for a small part lie in the reluctance of smokers to co-operate whereas the greater part of this increase must be due to one or more other factors.

A closer examination of Table VI reveals the following : With *lung cancer* a greatly increased death rate for smokers exists, which cannot be explained by the above-mentioned way of selection. This increased death rate from lung cancer—which disease accounts for 20 per cent of all deaths from all forms of cancer in persons aged 50–69—pushes up the death rate for " *all cancers* ", and Hammond and Horn's percentage of increase therefore considerably exceeds the percentage for the spurious increase as computed in the present paper. With " other cancers " this is not so, or only to a much smaller degree. But, whereas the importance of lung cancer compared with other forms of cancer greatly diminishes with advancing age, the importance of other forms of cancer, such as cancer of the larynx and the mouth, with which some kind of association with smoking may be suspected, greatly increases. This might explain part of the reversal in the percentages of increase in Column 6 of Table VI. Besides, we should not forget that in our highest age groups smoking habits differ a great deal from those of younger people.

Also with *coronary disease* Hammond and Horn found a considerable excess of the death rate for smokers. It should be stressed here that to conclude that there is a causal relationship between smoking and coronary disease, merely on the ground of this association, would be unwise. The same thing certainly applies to lung cancer. With lung cancer, however, there are a great number of facts implying causality ; with coronary disease this is not so or is so to a much smaller degree.

In " all causes " Hammond and Horn found a moderate increase. This is partly caused by two of the diseases included therein : lung cancer which shows a considerable excess but which amounts to only $3\frac{1}{2}$ per cent of all deaths, and coronary disease which shows a smaller excess but amounts to no less than 36 per cent of all deaths.

There remains, still, "other diseases" in which, without exception, our figures exceed those found by Hammond and Horn. This may indicate that our assumptions are a bit " overweighted ": that the percentage of co-operating smokers was higher than 65 per cent.

Berkson's and our assumption that the psychological reluctance of smokers to co-operate in this kind of investigation may cause a spurious increase in death rate for each separate cause of death, seems well founded. It is clear that the more heavily a person smokes, the greater will be this psychological reluctance as well as the spurious increase in the death rate caused by it. Therefore, that the association of deaths, from diseases other than lung cancer, with cigarette smoking is also shown in Hammond and Horn's study in a positive correlation between the death rate and the amount of smoking, does not astonish us at all. On the contrary, it is just what we expected. It confirms our opinion that this material does indeed bear the sign of selection by the elimination of many smokers, and it does not evidence in any way against the great probability of the importance of smoking as a causal factor in lung cancer—and perhaps in coronary disease.

Just as in this investigation, the death rates in Doll and Hill's prospective investigation are lower than the rates given by the official mortality statistics. The reason for this was believed to be the same as is discussed in our Section I.

In contrast with the findings of Hammond and Horn (1954), Doll and Hill (1954) found no excess of death rates for death from " other diseases " and from " cancer other than lung cancer ", and only a small excess for death from " coronary disease ". This seems to indicate that among their smokers only little reluctance to co-operate existed, which would be in keeping with the fact that the percentage of smokers in their study, and the percentage of smokers found in a social survey of Greater London, are almost the same.

The findings in Doll and Hill's investigation, therefore, seem to support the view on the significance of this selection in prospective studies on smoking and lung cancer, as expressed in this paper.

### SUMMARY AND CONCLUSIONS

Berkson (1955) points out, quite rightly, that, inasmuch as the death rates found by Hammond and Horn (1954) are lower than the death rates of the official mortality statistics, their material should be considered as " selected " with respect to the entire American population.

Berkson also points out, quite rightly, that, in research into an association between smoking and lung cancer, due attention should be given to the psychological reluctance of smokers to co-operate. By means of a " model ", based on a number of assumptions, he proves that this reluctance may lead to distortion of the result of investigations like Hammond and Horn's.

Starting from similar assumptions and using a similar model, it appeared possible to us, to fix, approximately, the size of this distortion. As a result of this investigation we found :

(1) that because of the action of this selection, as caused by the tendency for many smokers to eliminate themselves, only a small part of the excess in death rates for lung cancer and for coronary disease with smokers can be explained as being spurious ; therefore, the major part of this excess is not due to this selection but to one or more other fractors ;

(2) that this selection gives a plausible explanation for the excess in death rates for smokers for " other diseases " and " other cancers ", so that the excess in death rates in these diseases should be considered as being wholly artificial ;

(3) that the spurious excess in death rates for smokers, as caused by this selection, is proportional to the quantity of cigarettes smoked ;

(4) that, when in the reference population the percentage of smokers is assumed as being 80, this selection causes a decrease of this percentage to 72·5, so that part of the " shortage " of smokers, found in Hammond and Horn's investigation, can be explained by the action of this selection.

If our opinion is correct, it follows that Hammond and Horn's death rates entitle us to believe in a large and true excess of death rates with smoking in lung cancer and in coronary disease, whereas the excess they recorded in " other cancers " and in " other diseases " should be regarded as spurious.

To conclude :

Two objections which are often raised against Hammond and Horn's results : (1) the excess in death rates among smokers from causes of death with which an association with smoking would seem very improbable, and (2) the small percentage of cigarette smokers, may quite plausibly be explained by the psychological reluctance of smokers to enter this kind of investigation.

If we accept this psychological reluctance as a fact, Berkson's suggestion does not undermine—on the contrary it supports—our belief that cigarette smoking is causally related to lung cancer, and perhaps to coronary disease.

### APPENDIX

If we assume that, as regards death rates, Hammond and Horn's (1954) reference population is representative of the entire population of the U.S.A. their deficit of deaths from all causes in those aged 50–54 does not equal the difference between 1206 and 837, i.e. 369 (Table I). The reason is that Hammond and Horn's 100,000 persons were a selected group, owing to the elimination from the reference group of an unknown number of seriously ill persons—which elimination was responsible for the deficit of deaths.

We can make the following assumptions : (1) all of those who were eliminated died in the period under review—in this case during the next 20 months ; (2)

only half of these persons died during this period. The number of these persons will actually lie somewhere between both possibilities; probably nearer to the former than to the latter.

Let us first base our calculations on the former assumption. Suppose that, in order to reach their 837 deaths per 100,000 (Table I), Hammond and Horn had unknowingly eliminated $X$ deaths. This being so, their reference population would have numbered $100{,}000 + X$. The number of deaths they should have found in these $100{,}000 + X$ persons would be :

$$\left(100{,}000 \times \frac{837}{100{,}000}\right) + X$$

According to the official statistics the number of deaths in these $100{,}000 + X$ were :

$$(100{,}000 + X) \times \frac{1206}{100{,}000}$$

As in both cases the number of deaths is the same, we find : $X = 374$.

If we base our calculations on the second assumption, we find : $X = 378$. The difference between these two figures is minimal. We have based our calculations on the first assumption, from which it follows that, as regards death rates, the reference population from which each 100,000 of Hammond and Horn's age group 50–54 were selected, consisted of 100,374 persons.

For the separate causes of death the deficit in those aged 50–54 can be computed with the help of the following formula :

$$\text{deficit} = \frac{P \times 100{,}374}{100{,}000} - Q$$

in which $P$ means the official death rate per 100,000, and $Q$ the death rate per 100,000 as found by Hammond and Horn.

### REFERENCES

Berkson, J.—(1955) *Proc. Mayo Clin.*, **30**, 319.
Doll, R. and Hill, A. B.—(1954) *Brit. med. J.*, i, 1451.
Hammond, E. C. and Horn, D.—(1954) *J. Amer. med. Ass.*, **155**, 1316.
U.S. Dept. of Health.—(1954) Vital Statistics-Special Reports : National Summarie **40**, 57.